# Adherence to higher Life’s Essential 8 scores is linearly associated with reduced all-cause and cardiovascular mortality among US adults with metabolic syndrome: Results from NHANES 2005–2018

**DOI:** 10.1371/journal.pone.0314152

**Published:** 2024-11-22

**Authors:** Dao-Cheng Zhou, Jia-Lin Liang, Xin-Yu Hu, Hong-Cheng Fang, De-Liang Liu, Heng-Xia Zhao, Hui-Lin Li, Wen-Hua Xu

**Affiliations:** 1 Shenzhen Hospital of Integrated Traditional Chinese and Western Medicine, Shenzhen, Guangdong, China; 2 The Fourth Clinical Medical College of Guangzhou University of Chinese Medicine, Shenzhen, Guangdong, China; 3 Shenzhen Traditional Chinese Medicine Hospital, Shenzhen, Guangdong, China; Seth GS Medical College and KEM Hospital: King Edward Memorial Hospital and Seth Gordhandas Sunderdas Medical College, INDIA

## Abstract

**Background:**

Life’s Essential 8 (LE8) is the American Heart Association (AHA)’s recently updated assessment of cardiovascular health (CVH). Metabolic syndrome (MetS) is one of the most common chronic noncommunicable diseases associated with CVH impairment and an increased risk of mortality. However, the association of LE8 with all-cause and disease-specific mortality in the MetS population remains unknown. We aimed to explore these associations in a national prospective cohort study from NHANES 2005–2018.

**Methods:**

The LE8 was calculated according to the assessment criteria proposed by the AHA, which includes health behavior and health factor domains. LE8 scores were categorized as low CVH (0–49), moderate CVH (50–79), and high CVH (80–100). MetS was assessed according to NCEP-ATP III criteria, and mortality data were obtained through prospective linkage to the National Death Index database.

**Results:**

7839 participants with MetS were included and only 3.5% were in high CVH. In the fully adjusted models, LE8 was negatively associated with both all-cause and cardiovascular disease (CVD) mortality (hazard ratios [HR] and 95% confidence intervals [CI] of 0.978 (0.971,0.984) and 0.972 (0.961,0.984), respectively, both p < 0.0001). Both moderate/high CVH were associated with significantly lower mortality compared to low CVH (both p for trend <0.0001). Health behaviors had a more dominant effect compared to health factors. All-cause and CVD mortality gradually decreased with increasing ideal LE8 metrics. LE8 was not significantly associated with cancer mortality. LE8 and health behaviors were linearly associated with all-cause and CVD mortality, whereas health factors were nonlinearly associated (plateaued after ≥50). Education and chronic kidney disease influenced the association of LE8 with all-cause and CVD mortality, respectively.

**Conclusions:**

LE8 scores were negatively associated with all-cause and CVD mortality in the MetS population, while health behaviors had a dominant role. Adherence to higher CVH contributes to the prevention of excessive all-cause and CVD mortality in the MetS population.

## 1 Introduction

Metabolic syndrome (MetS) refers to a collective term for a group of cardiometabolic risk factors including abdominal obesity, insulin resistance, atherogenic dyslipidemia, and hypertension [1]. Due to the prevalence of unhealthy diets and lifestyles, MetS is now one of the most common chronic non-communicable conditions, affecting approximately one in four people globally [2]. The prevalence of MetS in the U.S. has increased significantly over the past few decades, and about one-third of adults are currently affected by MetS [3]. As a major global public health concern, MetS is strongly associated with an increased risk of various other major non-communicable diseases, including cardiovascular disease (CVD), type 2 diabetes (T2D), and cancers [4–6]. In addition, MetS significantly increased premature mortality compared with healthy populations. A national cohort study demonstrated that MetS was associated with significantly increased all-cause (hazard ratio [HR] = 1.24, 95% confidence interval [CI] = 1.16–1.33), heart disease (HR = 1.44, 95% CI = 1.25–1.66), and diabetes-related mortality (HR = 5.15, 95% CI = 3.15–8.43) [7]. CVD is a common complication in the MetS population and is the leading cause of morbidity and mortality [8]. A previous large meta-analysis demonstrated that MetS was associated with a significantly increased risk of CVD (relative risk [RR] = 2.35), CVD mortality (RR = 2.40), and all-cause mortality (RR = 1.58) [9].

Metabolic disorders such as insulin resistance and obesity in the MetS population are known to closely interact with the cardiovascular system and significantly impair cardiovascular health (CVH) [10], and suboptimal CVH has a profound impact on morbidity and mortality. Therefore, given the clinical prognostic relevance of CVD in MetS, maintaining a favorable CVH status may contribute to excess mortality prevention in the MetS population. In 2022, the American Heart Association (AHA) proposed a new metric for measuring and evaluating CVH, the Life’s Essential 8 (LE8) score [11]. LE8 integrates health behaviors and health factors to comprehensively assess CVH and is intended to shift the focus from disease treatment alone to health promotion for individuals and populations [11]. Since the publication of LE8, a large body of epidemiologic evidence has demonstrated that adherence to LE8-assessed CVH is associated with a reduced risk of developing CVD and its specific types [12–14]. In addition, higher LE8 scores were also associated with lower all-cause mortality and/or disease-specific mortality in various populations, such as the general population, people with chronic kidney disease (CKD), and cancer survivors [15–17]. However, whether maintaining good CVH status is associated with reduced all-cause and cause-specific mortality in the MetS population remains unclear. Addressing the association between LE8 scores and mortality in the MetS population may offer a deeper understanding of the impact of CVH on the prognosis of people with MetS and provide a scientific basis for developing targeted prevention strategies.

In this study, we utilized a national prospective cohort from the National Health and Nutrition Examination Survey (NHANES) to investigate the impact of LE8 and varying CVH statuses on all-cause, CVD, and cancer-related mortality in the MetS population, as well as to examine the respective effects of health behaviors and health factors. These findings may provide new insights into risk assessment and management of mortality in the MetS population. Our findings emphasize that adherence to a higher CVH status can contribute to the prevention of excess mortality in individuals with MetS and, in turn, reduce disease burden.

## 2 Methods

### Study design and population

NHANES is a principal epidemiologic program of the National Center for Health Statistics (NCHS) designed to assess the health and nutritional status of community-dwelling children and adults in the U.S. NHANES is an established, publicly available, free database comprising questionnaires and a range of medical and laboratory examination data. As of 1999, NHANES has been a continuous program of biennial cycles, with a nationally representative sample of approximately 5,000 cases per year. We obtained mortality outcomes prospectively by following up the baseline MetS population and therefore this was a prospective cohort study. All NHANES study protocols were approved by the NCHS Ethics Review Board (ERB) and informed consent was obtained from all participants and/or their legal guardian(s). The authors did not have access to information identifying individual participants during or after data collection. All methods in this study were performed in accordance with relevant guidelines and regulations.

**Fig 1:**
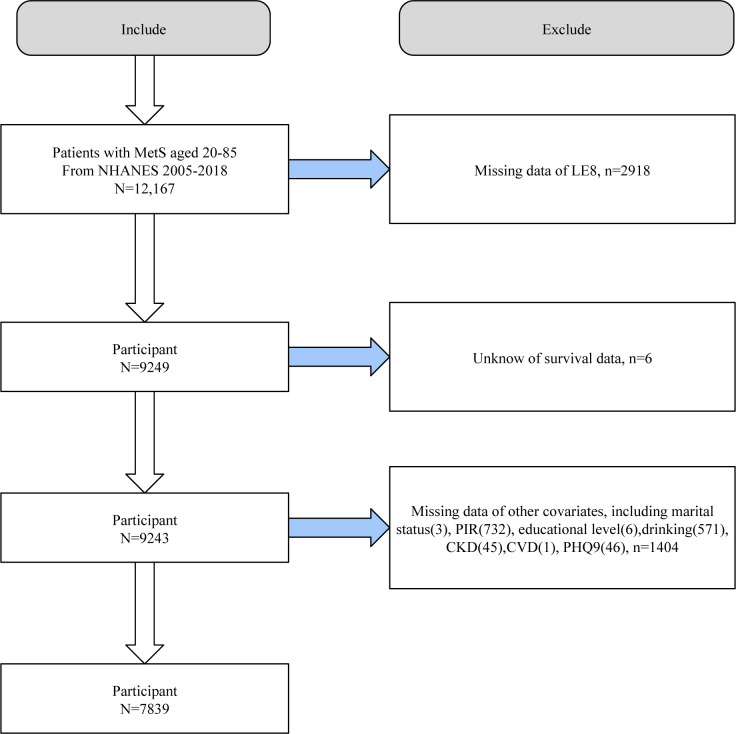
Flowchart of study population selection, NHANES 2005–2018.

We first included 12,167 adult MetS participants from NHANES 2005–2018. We then sequentially excluded participants with missing LE8 assessment information (n = 2918), survival data (n = 6), and covariates (n = 1404). 7839 eligible individuals with MetS were included in further analyses (**Fig 1**).

### Assessment of LE8 scores

The LE8 score was assessed according to the CVH components and assignment criteria proposed by the AHA [11], including 4 health behaviors and 4 health factors. The 4 health behaviors included diet, physical activity, nicotine exposure, and sleep health; and the 4 health factors included body mass index (BMI), blood lipids, blood glucose, and blood pressure (BP). Each component was assigned a score from 0–100, and the final LE8 score was obtained by dividing the score of each component by eight. Similarly, health behavior and health factor scores were averaged from the component scores in their respective domains. Based on LE8, health behavior, and health factor scores, participants were categorized as having low CVH (0–49 points), moderate CVH (50–79 points), and high CVH (80–100 points). In addition, each individual LE8 component ≥80 points was considered an ideal LE8 metric [18], and the number of ideal LE8 components achieved (0, 1, 2, 3, 4, 5, 6+) was also totaled and examined for potential dose-response trends.

Diet quality was assessed according to the Healthy Eating Index-2015 (HEI-2015), an index that evaluates the alignment of an individual’s food intake components with the Dietary Guidelines for Americans, including nine adequate intake components and four moderate intake components [19]. HEI-2015 was calculated based on intakes from two 24-h dietary recalls in NHANES and data from the USDA’s Food Patterns Equivalents Database [20]. Physical activity (PA) was assessed based on participants’ self-reported weekly moderate or vigorous physical activity (min/week) on the PAQ-K questionnaire in NHANES. Nicotine exposure was assessed according to self-reported history of smoking (cigarettes or other nicotine delivery systems) on the SMQ questionnaire. Sleep health was assessed based on participants’ self-reported average sleep duration per night. BMI is calculated based on the objectively measured weight (kg) divided by the square of the height (m) taken by trained staff at the Mobile Examination Center. Blood lipids (non-high-density lipoprotein cholesterol [non-HDL-C], specimens may be stored for 48 hours at 2°C-8°C before testing) and blood glucose (fasting blood glucose [FBG, fasting for 9 hours before the morning session] and HbA1c) were collected and measured by laboratory tests, and BP (systolic and diastolic blood pressure) was assessed by three consecutive measurements taken by specialized personnel at the Mobile Examination Center. The specific assignment criteria was presented in **S1 Table**.

### Assessment of MetS

In this study, MetS was assessed using the National Cholesterol Education Program-Adult Treatment Panel III criteria [21], which have been validated by a wealth of epidemiological studies, including NHANES-related studies [22]. The presence of MetS was indicated by meeting at least three of five criteria, including waist circumference (WC) ≥102 cm/88 cm for men and women, respectively; serum triglycerides (TG) ≥150 mg/dL, or drug treatment; serum HDL-C <40/50 mg/dL for men and women, respectively, or drug treatment; FBG ≥100 mg/dL or use of glucose-lowering medications; and BP ≥130/85 mm Hg or the use of antihypertensive therapy. In addition, in the sensitivity analysis, we used another widely used diagnostic criterion for MetS, the International Diabetes Federation (IDF) criteria [23].

### Mortality information collection

We obtained relevant all-cause and disease-specific mortality information by following the MetS population at baseline until December 31, 2019, and by prospectively matching records in the publicly available National Death Index database. CVD mortality information was drawn from codes associated with deaths from cardiac and cerebrovascular diseases, including ICD-10 codes I00-I09, I11, I13, I20-I51, and I60-I69, and cancer-related mortality was obtained from codes C00-C97.

### Covariates

We included multiple covariates that potentially could influence these associations to correct for confounding effects [15]. Important demographic variables including age, gender, race/ethnicity, educational attainment, household income-poverty ratio (PIR), and marital status were included. Other underlying lifestyle and disease states such as alcohol consumption, CVD history, CKD, and depression were also adjusted. Demographic variables were self-reported from the NHANES demographic file. Drinking history was categorized as never, former, and current light/moderate/heavy drinkers based on the NHANES Alcohol Use Questionnaire (ALQ) and prior studies [24]. CVD history was obtained from participants’ affirmative responses to questions on the NHANES MCQ questionnaire, and the presence of any of the following CVD types indicated the presence of CVD, including coronary heart disease/congestive heart failure/angina/stroke/heart attack. CKD was defined as a urine albumin/creatinine ratio ≥ 30 mg/g and/or an estimated glomerular filtration rate (eGFR) < 60 ml/min/1.73 m^2^ according to the KDIGO 2021 Clinical Practice Guideline [25]. The eGFR was calculated according to the widely accepted Chronic Kidney Disease Epidemiology Collaborative equation [26]. Depressive symptoms were assessed by the Patient Health Questionnaire-9 (PHQ-9), where a PHQ-9 ≥10 indicated the presence of major depression [27].

### Statistical analysis

Given the complex study design of NHANES, all analyses were appropriately weighted to obtain national estimates according to NHANES analytic guidelines. Data processing and statistical analyses were performed using R (version 4.2.3) and EmpowerStats, and statistical significance was defined as a P value of less than 0.05 (two-tailed). In the baseline analysis, we grouped the included MetS population according to CVH status assessed by LE8. Continuous variables were stated as mean ± standard error and tested for between-group differences using weighted analysis of variance; categorical variables were presented as number (percentage) and tested using weighted chi-square analysis. Kaplan-Meier (KM) survival analyses were performed to present survival curves for all-cause and cause-specific survival probabilities over time among different CVH statuses in the MetS population and to examine differences between groups using the log-rank test. Multivariate Cox proportional hazards regression models were applied to explore the association between LE8, health behaviors, and health factors scores and all-cause and disease-specific mortality in the MetS population. We constructed multiple models with varying degrees of adjustment, with the crude model not adjusting for any covariates; model 1 adjusting for age, sex, race/ethnicity; and model 2, the fully adjusted model, additionally adjusting for PIR, education level, marital status, alcohol consumption, history of CVD, CKD, and depression from model 1. Fully adjusted restricted cubic spline (RCS) models were used to explore potential nonlinear associations or linear associations (p for nonlinear < 0.05 suggests nonlinear association) [28]. Fully adjusted stratified analyses were employed to verify whether these associations remained stable across subgroups and to explore potential effect modifiers. Finally, we performed sensitivity analyses using IDF criteria to diagnose MetS to verify the consistency of the results. In addition, we excluded populations with CKD, CVD, cancer, or depression at baseline to rule out confounding effects from these major chronic noncommunicable diseases and to highlight the purpose of the study (association of LE8 with mortality in the MetS population).

## 3 Results

### Baseline characteristics

7839 participants with MetS were included with a mean age of 54.129 years (±0.265) and a mean LE8 score of 58.899. Only 274 (3.5%) of individuals with MetS were in high CVH. With increasing levels of CVH, MetS participants were younger, had higher PIR, LE8, health behaviors, health factors, and all eight LE8 components and respective scores, and were more likely to be of non-Hispanic White race/ethnicity, non-single, >high school educated, never/light/moderate drinkers, and people without depression, CKD, and CVD (**Table 1**).

**Table 1:** Baseline analysis of the MetS population according to CVH status, NHANES 2005–2018.

Variables	Total (n = 7839)	Low CVH (n = 2070)	Moderate CVH (n = 5495)	High CVH (n = 274)	P-value
**Age, year**	54.129±0.265	55.308±0.392	53.807±0.295	53.246±1.186	0.002
**PIR**	2.982±0.041	2.442±0.057	3.119±0.042	3.575±0.122	<0.0001
**LE8**	58.899±0.230	41.558±0.168	63.030±0.148	83.462±0.215	<0.0001
**Health Behaviors**	63.723±0.359	41.754±0.423	69.243±0.302	89.279±0.578	<0.0001
**Health Factors**	54.075±0.250	41.363±0.372	56.817±0.237	77.646±0.524	<0.0001
**HEI-2015**	52.726±0.241	46.303±0.322	54.008±0.264	66.625±0.747	**<0.0001**
**PA**	107.498±8.393	40.001±7.785	85.751±3.106	154.701±7.747	**<0.0001**
**Sleep duration**	7.104±0.025	6.715±0.048	7.208±0.029	7.442±0.064	**<0.0001**
**BMI**	33.206±0.104	36.145±0.217	32.558±0.104	28.049±0.339	**<0.0001**
**FBG**	120.290±0.916	134.904±2.103	116.796±0.864	105.712±1.239	**<0.0001**
**HbA1c**	5.986±0.019	6.553±0.039	5.841±0.017	5.383±0.031	**<0.0001**
**Non-HDL-C**	153.631±0.783	165.250±1.609	151.287±0.935	129.034±2.570	**<0.0001**
**SBP**	127.528±0.235	133.211±0.485	126.309±0.280	116.870±1.132	**<0.0001**
**DBP**	72.722±0.266	73.823±0.453	72.585±0.286	68.752±0.901	**<0.0001**
**smoking**					**<0.0001**
former	2392(31.131)	655(31.619)	1690(31.493)	47(21.166)	
never	3967(50.478)	603(27.801)	3137(56.074)	227(78.834)	
now	1480(18.391)	812(40.580)	668(12.433)	0 (0.000)	
**HEI-2015 diet score**	36.807±0.578	21.649±0.705	39.779±0.655	70.662±1.869	<0.0001
**Physical activity score**	64.935±0.706	29.232±1.357	74.393±0.728	97.006±0.846	<0.0001
**Nicotine exposure score**	70.747±0.601	46.992±1.284	76.944±0.572	93.954±1.007	<0.0001
**Sleep health score**	82.404±0.417	69.141±0.819	85.856±0.426	95.495±0.689	<0.0001
**Body mass index score**	38.385±0.452	25.968±0.715	40.796±0.502	66.581±2.222	<0.0001
**Blood lipids score**	52.284±0.470	41.522±0.911	54.574±0.578	72.832±1.879	<0.0001
**Blood glucose score**	71.732±0.497	55.512±0.779	75.660±0.503	93.464±1.461	<0.0001
**Blood pressure score**	53.901±0.449	42.449±0.748	56.237±0.543	77.705±1.725	<0.0001
**Sex**					0.125
male	3640(47.750)	960(45.497)	2581(48.683)	99(43.250)	
female	4199(52.250)	1110(54.503)	2914(51.317)	175(56.750)	
**Race/ethnicity**					<0.0001
Mexican American	1285(7.945)	284(7.156)	945(8.040)	56(10.864)	
Non-Hispanic Black	1393(8.348)	535(13.895)	840(6.909)	18(2.785)	
Non-Hispanic White	3913(74.265)	987(70.639)	2795(75.372)	131(74.665)	
Other Hispanic	717(4.317)	179(4.193)	512(4.350)	26(4.435)	
Other Race	531(5.125)	85(4.116)	403(5.329)	43(7.251)	
**Marital Status**					<0.0001
non-single	4906(67.533)	1166(60.865)	3528(68.907)	212(81.140)	
single	2933(32.467)	904(39.135)	1967(31.093)	62(18.860)	
**Education**					<0.0001
<high school	820(5.148)	254(7.421)	548(4.604)	18(1.969)	
high school	3214(38.675)	1010(48.750)	2130(36.194)	74(25.971)	
>high school	3805(56.178)	806(43.829)	2817(59.202)	182(72.061)	
**Drinking**					<0.0001
never	1150(11.421)	255(10.140)	840(11.677)	55(14.195)	
former	1770(19.105)	584(25.939)	1150(17.317)	36(12.532)	
mild	2594(37.411)	585(30.704)	1896(38.927)	113(48.500)	
moderate	1011(14.517)	248(13.831)	719(14.571)	44(17.621)	
heavy	1314(17.545)	398(19.387)	890(17.508)	26(7.153)	
**Depression**					<0.0001
No	6987(90.604)	1686(82.950)	5037(92.650)	264(97.115)	
Yes	852(9.396)	384(17.050)	458(7.350)	10(2.885)	
**CVD**					<0.0001
No	6364(84.112)	1510(75.102)	4612(86.512)	242(91.950)	
Yes	1475(15.888)	560(24.898)	883(13.488)	32(8.050)	
**CKD**					<0.0001
No	5650(77.074)	1282(66.734)	4132(79.689)	236(88.764)	
Yes	2189(22.926)	788(33.266)	1363(20.311)	38(11.236)	

Continuous variables were stated as mean ± standard error and tested for between-group differences using weighted analysis of variance; categorical variables were presented as number (percentage) and tested using weighted chi-square analysis.

### KM survival analysis

**Fig 2:**
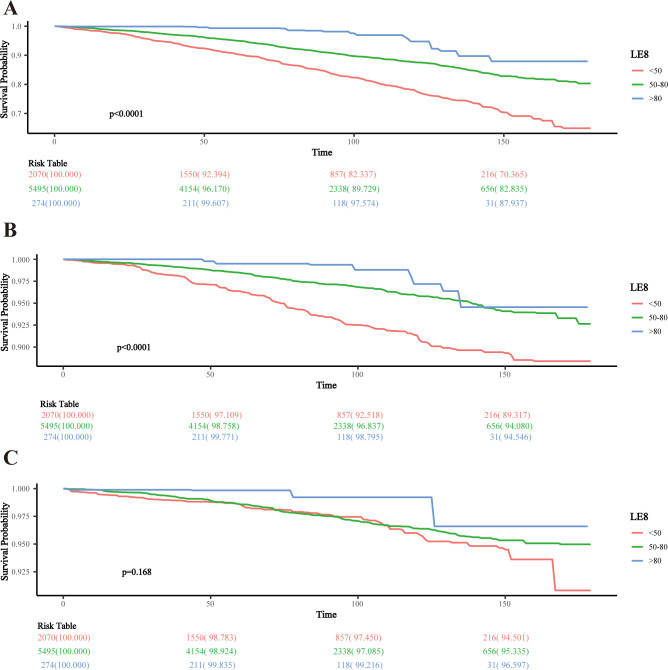
KM survival analysis of LE8 and mortality in the MetS population. A: All-cause; B: CVD; C: cancer.

The KM survival curves indicated significantly higher all-cause and CVD-related survival probabilities over time for MetS participants with moderate and high CVH status compared to low CVH (both log-rank p < 0.0001). However, there was no significant difference in cancer-related survival probabilities across CVH status (p = 0.168) (**Fig 2**).

### Association of LE8 with mortality in the MetS population

**Fig 3:**
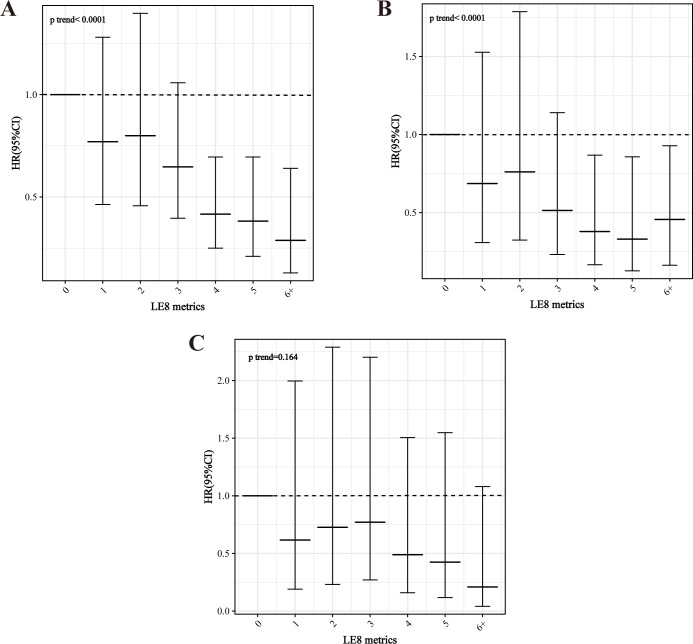
Association of the number of ideal LE8 metrics with mortality in the MetS population. A: All-cause; B: CVD; C: cancer.

After a median of 87 months (interquartile range, 50–128 months), 1,138 MetS participants died, with 392 and 255 CVD- and cancer-related deaths, respectively. After adjusting for all confounders, LE8 and health behaviors scores remained negatively associated with all-cause mortality (LE8: HR = 0.978, 95% CI = 0.971–0.984, p<0.0001; health behaviors: HR = 0.985, 95% CI = 0.981–0.988, p<0.0001), whereas health factors scores lost their association (p = 0.074). Compared to low CVH, being at moderate/high CVH for both LE8 and health behaviors was associated with significantly lower all-cause mortality (LE8: HRs of 0.637 and 0.314 for moderate and high CVH, respectively; and health behaviors: HRs of 0.723 and 0.477 for moderate and high CVH, respectively; both p for trend < 0.0001) (**Table 2**). Similarly, LE8 and health behaviors remained associated with CVD mortality in the MetS population in fully adjusted models (LE8: HR and 95% CI = 0.972 (0.961,0.984), p < 0.0001; health behaviors: HR and 95% CI = 0.986 (0.978,0.993), p < 0.001). Of note, health factors scores were also significantly negatively associated with CVD mortality (HR and 95% CI = 0.986 (0.976,0.995), p = 0.004). Compared to low CVH, both LE8 and health behaviors scores at moderate/high CVH were associated with significantly lower CVD mortality, while health factors at moderate CVH were associated with lower CVD mortality (**Table 3**). However, after adjusting for all covariates, none of the LE8, health behaviors, and health factors were significantly associated with cancer mortality in the MetS population, except for the continuous health behaviors score (HR = 0.989, p = 0.013) (**S2 Table**). Similarly, all-cause and CVD mortality in the MetS population exhibited a dose-response decline with increasing ideal LE8 metrics (p for trend both <0.0001), whereas there was no significant association with cancer mortality (p for trend = 0.164) (**Fig 3**).

**Table 2:** Association of LE8, health behaviors, and health factors with all-cause mortality in the MetS population.

	Crude Model HR (95%CI)	P-value	Model 1 HR (95%CI)	P-value	Model 2 HR (95%CI)	P-value
**LE8**	0.972(0.966,0.978)	<0.0001	0.967(0.960,0.974)	<0.0001	**0.978(0.971,0.984)**	**<0.0001**
**LE8**						
**Low CVH**	ref	ref	ref	ref	ref	ref
**Moderate CVH**	0.525(0.446,0.617)	<0.0001	0.496(0.416,0.592)	<0.0001	**0.637(0.532,0.763)**	**<0.0001**
**High CVH**	0.224(0.125,0.402)	<0.0001	0.193(0.111,0.335)	<0.0001	**0.314(0.172,0.574)**	**<0.001**
**P for trend**	<0.0001		<0.0001		**<0.0001**	
**Health behaviors**	0.986(0.982,0.990)	<0.0001	0.978(0.975,0.982)	<0.0001	**0.985(0.981,0.988)**	**<0.0001**
**Health behaviors**						
**Low CVH**	ref	ref	ref	ref	ref	ref
**Moderate CVH**	0.716(0.602,0.853)	<0.001	0.578(0.497,0.672)	<0.0001	**0.723(0.609,0.858)**	**<0.001**
**High CVH**	0.461(0.360,0.592)	<0.0001	0.342(0.275,0.427)	<0.0001	**0.477(0.376,0.605)**	**<0.0001**
**P for trend**	<0.0001		<0.0001		**<0.0001**	
**Health factors **	0.983(0.978,0.989)	<0.0001	0.989(0.984,0.995)	<0.001	0.995(0.989,1.001)	0.074
**Health factors **						
**Low CVH**	ref	ref	ref	ref	ref	ref
**Moderate CVH**	0.661(0.568,0.769)	<0.0001	0.773(0.657,0.910)	0.002	0.890(0.748,1.059)	0.189
**High CVH**	0.574(0.370,0.890)	0.013	0.854(0.564,1.292)	0.454	1.040(0.676,1.600)	0.858
**P for trend**	<0.0001		0.005		0.334	

The crude model did not adjust for any covariates; model 1 adjusted for age, sex, race/ethnicity; and model 2 additionally adjusted for PIR, education level, marital status, alcohol consumption, history of CVD, CKD, and depression from model 1.

**Table 3:** Association of LE8, health behaviors, and health factors with CVD mortality in the MetS population.

	Crude Model HR (95%CI)	P-value	Model 1 HR (95%CI)	P-value	Model 2 HR (95%CI)	P-value
**LE8**	0.968(0.959,0.978)	<0.0001	0.961(0.949,0.973)	<0.0001	**0.972(0.961,0.984)**	**<0.0001**
**LE8**						
**Low CVH**	ref	ref	ref	ref	ref	ref
**Moderate CVH**	0.469(0.351,0.627)	<0.0001	0.439(0.322,0.597)	<0.0001	**0.566(0.415,0.772)**	**<0.001**
**High CVH**	0.281(0.122,0.647)	0.003	0.235(0.107,0.517)	<0.001	**0.381(0.164,0.881)**	**0.024**
**P for trend**	<0.0001		<0.0001		**<0.0001**	
**Health behaviors**	0.987(0.981,0.994)	<0.001	0.979(0.972,0.987)	<0.0001	**0.986(0.978,0.993)**	**<0.001**
**Health behaviors**						
**Low CVH**	ref	ref	ref	ref	ref	ref
**Moderate CVH**	0.698(0.537,0.907)	0.007	0.549(0.427,0.707)	<0.0001	**0.688(0.516,0.919)**	**0.011**
**High CVH**	0.532(0.350,0.808)	0.003	0.388(0.253,0.595)	<0.0001	**0.541(0.348,0.840)**	**0.006**
**P for trend**	0.002		<0.0001		**0.004**	
**Health factors **	0.975(0.967,0.983)	<0.0001	0.980(0.970,0.989)	<0.0001	**0.986(0.976,0.995)**	**0.004**
**Health factors **						
**Low CVH**	ref	ref	ref	ref	ref	ref
**Moderate CVH**	0.535(0.415,0.689)	<0.0001	0.627(0.476,0.825)	<0.001	**0.719(0.540,0.958)**	**0.024**
**High CVH**	0.435(0.213,0.890)	0.023	0.653(0.335,1.273)	0.211	0.809(0.408,1.604)	0.544
**P for trend**	<0.0001		0.001		**0.042**	

The crude model did not adjust for any covariates; model 1 adjusted for age, sex, race/ethnicity; and model 2 additionally adjusted for PIR, education level, marital status, alcohol consumption, history of CVD, CKD, and depression from model 1.

### RCS analysis

**Fig 4:**
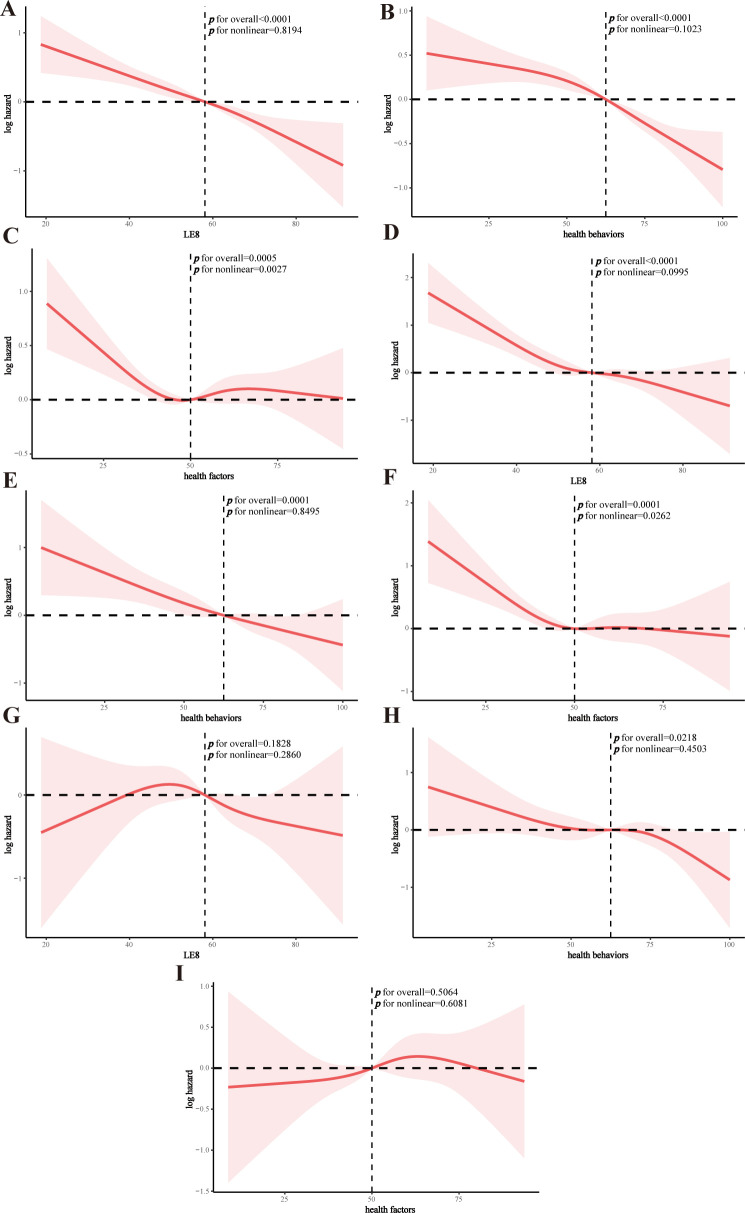
RCS analysis of the association of LE8, health behaviors, and health factors and mortality in the MetS population. A: LE8 and all-cause mortality; B: health behaviors and all-cause mortality; C: health factors and all-cause mortality; D: LE8 and CVD mortality; E: health behaviors and CVD mortality; F: health factors and CVD mortality; G: LE8 and cancer mortality; H: health behaviors and cancer mortality; I: health factors and cancer mortality.

The RCS model suggested that both LE8 and health behavior scores were linearly associated with all-cause mortality in the MetS population (p for nonlinear = 0.8194 and 0.1023, respectively), whereas health factors were nonlinearly associated with all-cause mortality (p for nonlinear = 0.0027) (**Fig 4A–4C**). Similar patterns were found for CVD mortality (**Fig 4D–4F**). LE8 and health factors were not associated with cancer mortality, while there was a linear association between health behaviors and cancer mortality (**Fig 4G–4I**). Threshold effect analyses demonstrated significant associations between health factors scores and all-cause and CVD mortality in the MetS population at <50 (all-cause: HR and 95% CI = 0.982 (0.968,0.996), p = 0.015; CVD: HR and 95% CI = 0.967 (0.947,0.989), p = 0.003), and lost associations after the inflection point (**Table 4**).

**Table 4:** Threshold effect analysis of health factors scores in relation to all-cause and CVD mortality in the MetS population.

	HR (95%CI) P-value	P-interaction
**All-cause mortality**		
**Health Factors<50**	**0.982(0.968,0.996) 0.015**	**0.048**
**Health Factors**≥**50**	1.003(0.991,1.015) 0.597	
**CVD mortality**		
**Health Factors<50**	**0.967(0.947,0.989) 0.003**	**0.0022**
**Health Factors**≥**50**	1.002(0.988,1.017) 0.758	

### Stratified analysis

**Fig 5:**
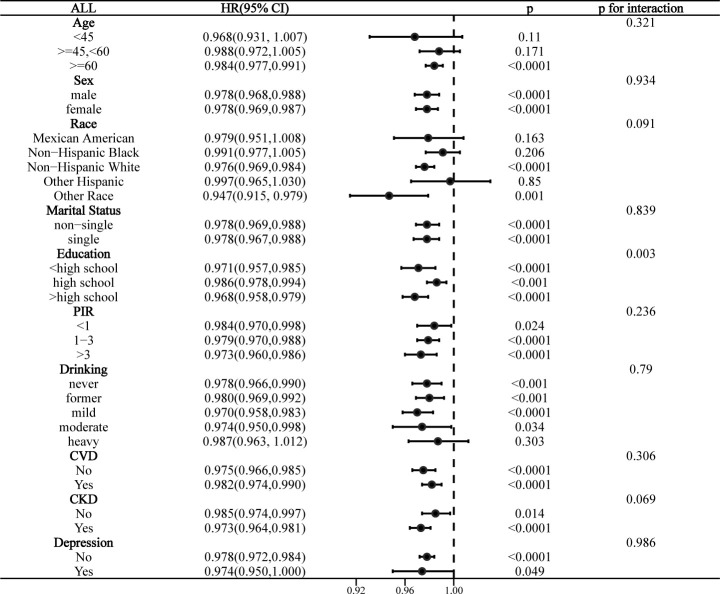
Stratified analysis of the association between LE8 and all-cause mortality in the MetS population.

**Fig 6:**
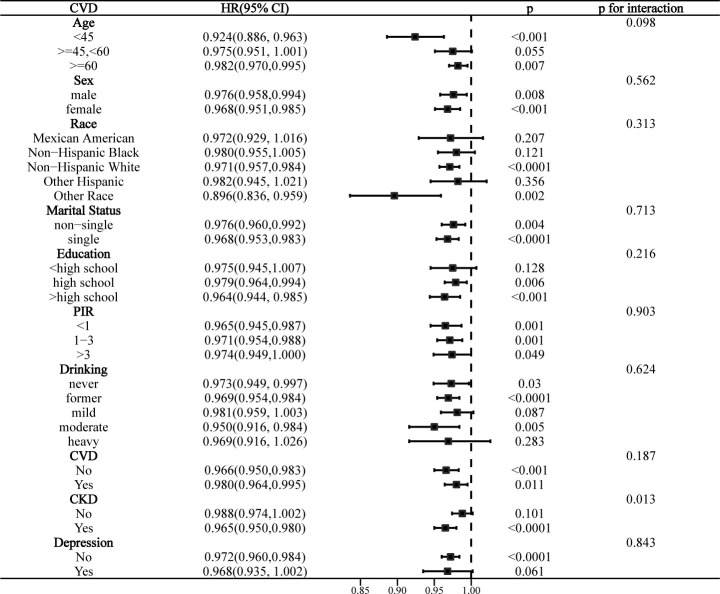
Stratified analysis of the association between LE8 and CVD mortality in the MetS population.

Interaction analyses indicated that education level significantly affected the association of LE8 with all-cause mortality in the MetS population (p for interaction = 0.003), and this association was more significant in those with >high school education (**Fig 5**). CKD significantly affected the association of LE8 with CVD mortality (p for interaction = 0.013), and this association was only present in the CKD population (**Fig 6**). Notably, while there were no statistically significant differences in the risk of all-cause mortality and CVD mortality by race (interaction p > 0.05). However, “other race” showed statistically significant differences in both stratified analyses with more pronounced effect sizes, suggesting that specific racial subgroups may have different mortality risks associated with LE8. The association between LE8 and cancer mortality was not affected by any covariate and was not significant in all subgroups (**S1 Fig**).

### Sensitivity analysis

The use of IDF criteria for diagnosing MetS yielded largely consistent findings. LE8 and health behaviors remained significantly associated with all-cause and CVD mortality. Compared to low CVH, health factors were associated with all-cause and CVD mortality only at moderate CVH. LE8, health behaviors, and health factors were all not associated with cancer mortality (**S3–S5 Tables**). Consistently, exclusion of participants with CKD, CVD, cancer, or depression at baseline did not significantly change the primary findings. In fully adjusted model 2, LE8 and health behaviors remained negatively associated with all-cause mortality in the MetS population (LE8: HR 0.983, p = 0.015; health behaviors: HR 0.985, p = 0.013), whereas health factors were similarly unrelated (p = 0.318) (**S6 Table**). LE8, health behaviors, and health factors remained negatively associated with CVD mortality in the MetS population (LE8: HR 0.967, p < 0.0001; health behaviors: HR 0.984, p < 0.001; health factors: HR 0.980, p < 0.001) (**S7 Table**). Similarly, only health behaviors were negatively associated with cancer mortality in the MetS population (HR of 0.985, p = 0.002) (**S8 Table**).

## 4 Discussions

In this large, nationally representative prospective cohort study, our findings demonstrated for the first time that the LE8 scores proposed by the AHA were significantly negatively associated with all-cause and CVD mortality in the MetS population, with health behaviors having a more dominant role. Consistently, adherence to more ideal LE8 metrics was associated with progressively lower all-cause and CVD mortality in the MetS population. LE8 was not significantly associated with cancer mortality. There were dose-response associations of LE8 and health behaviors with both all-cause and CVD mortality, while health factors were nonlinearly associated. Education level and CKD significantly influenced the association of LE8 with all-cause and CVD mortality, respectively.

To the best of our knowledge, there is still a dearth of research investigating the public health significance of LE8 scores and their assessed CVH in MetS populations. Only one recent cross-sectional study similarly using NHANES has explored the association between LE8 and the prevalence of MetS in the general population, and unsurprisingly showed that higher LE8 scores were associated with significantly lower odds of MetS [29]. Another cross-sectional analysis using NHANES suggested that LE8 scores were negatively associated with the prevalence of metabolic unhealth (MUH) in the general population, and that each 10-point increase in LE8 scores was associated with a 47% reduction in the odds of MUH [30]. Although they were both cross-sectional analyses, these real-world studies suggested a close clinical association between CVH assessed by LE8 and the development of MetS, emphasizing the crosstalk between metabolic disorders and the cardiovascular system and some shared pathogenic mechanisms, including insulin resistance, systemic inflammation, and oxidative stress [8, 31].

There are no studies exploring the association between LE8 scores and mortality in MetS populations. However, cumulative evidence from longitudinal cohort studies has suggested that higher LE8 scores are associated with reduced mortality in both general and disease-specific populations. Sun et al. used data from NHANES 2005–2014 to show that compared to low CVH, general populations at both moderate and high CVH were associated with significantly lower all-cause and CVD mortality, with dose-response associations [32]. Yi et al. obtained similar conclusions using NHANES 2005–2014, demonstrating that higher LE8 scores were inversely associated with all-cause and CVD mortality in the general U.S. adult population (HRs of 0.86 and 0.81, respectively) [15]. A prospective cohort study utilizing NHANES 2005–2018 demonstrated that each 10-point increase in LE8 was associated with 17%, 12%, and 18% reductions in all-cause, CVD, and cancer mortality, respectively, in the CKD population [16]. Another prospective cohort study using NHANES 2007–2018 demonstrated an inverse dose-response association of LE8 with all-cause and CVD mortality in cancer survivors, but not with cancer mortality [17]. Other prospective cohort studies have also demonstrated that LE8 scores were significantly associated with all-cause mortality in stroke survivors, individuals with CVD, and rheumatoid arthritis populations [33–35].

Notably, several studies have shown a significant association between LE8 and mortality in people with other metabolic diseases. In a prospective cohort study similarly from NHANES, Shen et al. demonstrated that higher LE8 scores were associated with significantly lower all-cause and CVD mortality in the T2D population (all-cause mortality: HR = 0.71, 95% CI = 0.62–0.81; CVD mortality: HR = 0.68, 95% CI = 0.58–0.85) [36]. Sun et al. used data from the UK Biobank in a prospective cohort study to demonstrate that both moderate and high CVH status were associated with reduced all-cause mortality in both T2D and non-T2D populations compared to low CVH, and that being in high CVH significantly increased the predicted life expectancy of participants (at age 50 years) [37]. Interestingly, adherence to the LE8 score provided a significantly higher mortality prevention benefit in the T2D population than in the non-T2D population [37]. A recent prospective cohort study utilizing NHANES 2005–2018 demonstrated that each 10-point increase in LE8 scores in people with insulin resistance was associated with 15% and 31% reductions in all-cause and CVD mortality, respectively, and that systemic inflammation and vascular aging partially mediated these associations [38]. Our findings were consistent with these studies showing that higher LE8 scores were associated with reduced all-cause and CVD mortality in the MetS population, providing population-level prospective evidence for mortality risk assessment, stratification, and management of the LE8 in the MetS population. However, we observed that LE8 scores were not associated with reduced cancer mortality in the MetS population. A previous study similarly showed that LE8 was not associated with cancer mortality in cancer survivors [17]. A previous prospective cohort study utilizing NHANES demonstrated that MetS was not associated with cancer mortality (HR = 1.17, 95% CI = 0.95–1.43) [7]. The “obesity paradox” suggested by previous literature may be a possible explanation for this phenomenon [39–41]. People who are obese or have a higher BMI perform better than normal weight individuals in terms of survival outcomes for certain chronic diseases, including advanced cancer and CVD [42–45]. This paradox is equally likely to exist in our study population (MetS). The reason for this paradoxical effect may be that excess body weight serves as a metabolic reserve during illness [46], especially in the elderly or frail, which may confound the benefits of improved LE8 scores in reducing cancer mortality. In addition, uncontrolled confounding factors and reverse causation may exist [47]. Reverse causation occurs frequently in studies of cancer and CVD, which may have led to similar results in the MetS population of this study. The relationship between LE8 and cancer-related mortality in the MetS population needs to be further validated in future large prospective studies. In conclusion, although the underlying mechanisms of how LE8 affects mortality in MetS populations remain unexplored, based on previous studies, we hypothesized that systemic inflammation, oxidative stress, and vascular aging may contribute to the underlying mechanisms.

A growing body of epidemiologic evidence suggests that adherence to a healthy lifestyle is the cornerstone of clinical management of MetS populations and is associated with mortality prevention. Niu et al. demonstrated using NHANES 2007–2014 that adherence to an emerging composite lifestyle score (including smoking, alcohol consumption, physical activity, diet, sleep duration, and sedentary) was associated with a significant reduction in all-cause mortality in the MetS population [48]. Higher diet quality and physical activity participation have been shown to be associated with lower all-cause mortality in the MetS population [49, 50]. The health factor domains in LE8, on the other hand, have some overlap with the components of MetS [30], and maintaining better metabolic health may reduce MetS severity and thus improve prognosis [51]. However, we found that the association of health factors with mortality in the MetS population was significantly weaker than that of health behaviors, suggesting the dominant role of health behaviors for MetS mortality prevention. Specifically, we found nonlinear associations of health factor scores with all-cause and CVD mortality in the MetS population, which were negatively associated with mortality within a certain range and flattened after the inflection point. Thus, maintaining an appropriate level of metabolic fitness rather than over-achieving a healthy metabolic profile may be more conducive to mortality prevention.

We found that education level and CKD status significantly influenced the association between LE8 and all-cause and CVD mortality in the MetS population, respectively. Some previous clinical studies have similarly shown that the association of LE8 with specific diseases is influenced by education [16, 52]. There were significant socioeconomic status differences in the distribution of LE8, which was significantly higher in the highly educated population [53, 54]. Thus, this finding suggests that adherence to LE8 scores has better mortality prevention value in MetS populations with greater than high school education, suggesting the need for individualized CVH assessment strategies. A close inter-crosstalk between CKD, MetS, and CVD has been demonstrated [55]. Our study suggests that the protective effect of LE8 on CVD mortality exists only in the CKD population. CKD coexistence may adversely affect clinical outcomes in the MetS population [56]. Thus, adherence to LE8 scores may have more significant preventive value in the CKD population, suggesting a focus on the CVH status of the MetS and CKD comorbid population and the value of risk assessment and management provided by their LE8 in clinical practice.

Our study has some significant advantages. It was a large sample, nationally representative cohort study from NHANES with good generalizability and replicability. The prospective nature of the study and well-considered adjustment for confounders made the findings reliable and reduced study bias. This study has important public health implications in that it provides up-to-date population-level evidence for mortality risk assessment and management with the LE8 in the MetS population and provides underpinning for adherence to a higher CVH for mortality prevention. However, there are limitations to our study. Some LE8 components were based on participant self-report and therefore may be subject to recall bias. However, numerous previous NHANES-related studies have demonstrated the good reliability of these standardized AHA-based assessment methods. Second, although our study utilized a nationally representative cohort from the NHANES database, the generalizability of the findings may be limited by the composition of the study population. In the future, we emphasize the need to further validate our findings in a broader ethnic group. In addition, we were unable to assess the effect of longitudinal changes in LE8 on mortality in the MetS population. Finally, limited by the nature of observational studies, we were unable to draw causal associations. Future well-designed large-scale prospective studies are needed to validate our findings in other populations and to further reveal the clinical utility of LE8.

## 5 Conclusions

In a national prospective cohort study, LE8 scores were negatively associated with all-cause and CVD mortality in the MetS population, with health behaviors having a more dominant role. LE8 was not significantly associated with cancer mortality. LE8 and health behaviors had dose-response associations with all-cause and CVD mortality, whereas health factors were nonlinearly associated, suggesting the need for moderate adjustment of health factors. Education and CKD significantly influenced these associations. These findings suggest that adherence to a higher CVH contributes to all-cause and CVD mortality prevention in the MetS population and support mortality risk assessment and management through LE8 evaluation.

## Supporting information

S1 Table Definition and scoring approach for the American Heart Association’s LE8 score. (DOCX)

S2 Table Association of LE8, health behaviors, and health factors with cancer mortality in the MetS population. The crude model did not adjust for any covariates; model 1 adjusted for age, sex, race/ethnicity; and model 2 additionally adjusted for PIR, education level, marital status, alcohol consumption, history of CVD, CKD, and depression from model 1.
(DOCX)

S3 Table Association of LE8 with all-cause mortality in the IDF-MetS population. The crude model did not adjust for any covariates; model 1 adjusted for age, sex, race/ethnicity; and model 2 additionally adjusted for PIR, education level, marital status, alcohol consumption, history of CVD, CKD, and depression from model 1.
(DOCX)

S4 Table Association of LE8 with CVD mortality in the IDF-MetS population. The crude model did not adjust for any covariates; model 1 adjusted for age, sex, race/ethnicity; and model 2 additionally adjusted for PIR, education level, marital status, alcohol consumption, history of CVD, CKD, and depression from model 1.
(DOCX)

S5 Table Association of LE8 with cancer mortality in the IDF-MetS population. The crude model did not adjust for any covariates; model 1 adjusted for age, sex, race/ethnicity; and model 2 additionally adjusted for PIR, education level, marital status, alcohol consumption, history of CVD, CKD, and depression from model 1.
(DOCX)

S6 Table Association of LE8, health behaviors, and health factors with all-cause mortality in the MetS population after excluding participants with chronic kidney disease, cardiovascular disease, cancer, or depression at baseline. (DOCX)

S7 Table Association of LE8, health behaviors, and health factors with CVD mortality in the MetS population after excluding participants with chronic kidney disease, cardiovascular disease, cancer, or depression at baseline. (DOCX)

S8 Table Association of LE8, health behaviors, and health factors with cancer mortality in the MetS population after excluding participants with chronic kidney disease, cardiovascular disease, cancer, or depression at baseline. Model 1: Age, Sex, Race. Model 2: Age, Sex, Race, PIR, marital status, education, drinking.
(DOCX)

S1 Fig Stratified analysis of the association between LE8 and cancer mortality in the MetS population. (DOCX)

S1 Raw data (CSV)
