# Supplementary material for: Adherence to higher Life’s Essential 8 scores is linearly associated with reduced all-cause and cardiovascular mortality among US adults with metabolic syndrome: Results from NHANES 2005–2018
Source: PLoS One. 2024 Nov 22;19(11):e0314152. doi: 10.1371/journal.pone.0314152 (PMC11584117; doi:10.1371/journal.pone.0314152)
Supplement: S2 Table — The crude model did not adjust for any covariates; model 1 adjusted for age, sex, race/ethnicity; and model 2 additionally adjusted for PIR, education level, marital status, alcohol consumption, history of CVD, CKD, and depression from model 1. (DOCX) [file pone.0314152.s002.docx]

**S2 Table. Association of LE8, health behaviors, and health factors with cancer mortality in the MetS population.**

|  | **Crude Model**  **HR (95%CI)** | **P-value** | **Model 1**  **HR (95%CI)** | **P-value** | **Model 2**  **HR (95%CI)** | **P-value** |
| --- | --- | --- | --- | --- | --- | --- |
| **LE8** | 0.987(0.976,0.999) | 0.033 | 0.984(0.971,0.997) | 0.018 | 0.991(0.977,1.005) | 0.215 |
| **LE8** | | | | | | |
| **Low CVH** | ref | ref | ref | ref | ref | ref |
| **Moderate CVH** | 0.816(0.562,1.185) | 0.286 | 0.773(0.523,1.142) | 0.196 | 0.895(0.591,1.357) | 0.603 |
| **High CVH** | 0.402(0.107,1.510) | 0.177 | 0.351(0.093,1.325) | 0.123 | 0.466(0.116,1.880) | 0.283 |
| **P for trend** |  | 0.11 |  | 0.059 |  | 0.342 |
| **health behaviors** | 0.991(0.984,0.999) | 0.027 | 0.985(0.976,0.993) | <0.001 | 0.989(0.980,0.998) | 0.013 |
| **health behaviors** | | | | | | |
| **Low CVH** | ref | ref | ref | ref | ref | ref |
| **Moderate CVH** | 0.973(0.694,1.364) | 0.872 | 0.796(0.563,1.126) | 0.197 | 0.933(0.640,1.360) | 0.717 |
| **High CVH** | 0.707(0.432,1.157) | 0.168 | 0.535(0.331,0.865) | 0.011 | 0.676(0.411,1.112) | 0.124 |
| **P for trend** |  | 0.162 |  | 0.008 |  | 0.119 |
| **health factors** | 0.996(0.986,1.007) | 0.506 | 1.003(0.991,1.015) | 0.619 | 1.006(0.994,1.018) | 0.353 |
| **health factors** | | | | | | |
| **Low CVH** | ref | ref | ref | ref | ref | ref |
| **Moderate CVH** | 0.984(0.706,1.370) | 0.922 | 1.130(0.809,1.579) | 0.473 | 1.216(0.861,1.717) | 0.267 |
| **High CVH** | 0.948(0.438,2.052) | 0.892 | 1.407(0.644,3.078) | 0.392 | 1.553(0.706,3.418) | 0.274 |
| **P for trend** |  | 0.878 |  | 0.313 |  | 0.156 |

The crude model did not adjust for any covariates; model 1 adjusted for age, sex, race/ethnicity; and model 2 additionally adjusted for PIR, education level, marital status, alcohol consumption, history of CVD, CKD, and depression from model 1.
