# Supplementary material for: Adherence to higher Life’s Essential 8 scores is linearly associated with reduced all-cause and cardiovascular mortality among US adults with metabolic syndrome: Results from NHANES 2005–2018
Source: PLoS One. 2024 Nov 22;19(11):e0314152. doi: 10.1371/journal.pone.0314152 (PMC11584117; doi:10.1371/journal.pone.0314152)
Supplement: S3 Table — The crude model did not adjust for any covariates; model 1 adjusted for age, sex, race/ethnicity; and model 2 additionally adjusted for PIR, education level, marital status, alcohol consumption, history of CVD, CKD, and depression from model 1. (DOCX) [file pone.0314152.s003.docx]

**S3 Table. Association of LE8 with all-cause mortality in the IDF-MetS population.**

|  | **Crude Model**  **HR (95%CI)** | **P-value** | **Model 1**  **HR (95%CI)** | **P-value** | **Model 2**  **HR (95%CI)** | **P-value** |
| --- | --- | --- | --- | --- | --- | --- |
| **LE8** | 0.972(0.966,0.979) | <0.0001 | 0.966(0.959,0.973) | <0.0001 | 0.977(0.971,0.984) | <0.0001 |
| **LE8** | | | | | | |
| **Low CVH** | ref | ref | ref | ref | ref | ref |
| **Moderate CVH** | 0.516(0.437,0.610) | <0.0001 | 0.483(0.405,0.577) | <0.0001 | 0.617(0.516,0.738) | <0.0001 |
| **High CVH** | 0.249(0.137,0.452) | <0.0001 | 0.209(0.120,0.365) | <0.0001 | 0.334(0.181,0.617) | <0.001 |
| **P for trend** |  | <0.0001 |  | <0.0001 |  | <0.0001 |
| **health behaviors** | 0.987(0.983,0.991) | <0.0001 | 0.979(0.975,0.982) | <0.0001 | 0.985(0.981,0.989) | <0.0001 |
| **health behaviors** | | | | | | |
| **Low CVH** | ref | ref | ref | ref | ref | ref |
| **Moderate CVH** | 0.762(0.632,0.918) | 0.004 | 0.601(0.511,0.705) | <0.0001 | 0.750(0.624,0.901) | 0.002 |
| **High CVH** | 0.473(0.361,0.618) | <0.0001 | 0.340(0.271,0.426) | <0.0001 | 0.468(0.371,0.591) | <0.0001 |
| **P for trend** |  | <0.0001 |  | <0.0001 |  | <0.0001 |
| **health factors** | 0.982(0.976,0.987) | <0.0001 | 0.988(0.982,0.993) | <0.0001 | 0.993(0.988,0.999) | 0.013 |
| **health factors** | | | | | | |
| **Low CVH** | ref | ref | ref | ref | ref | ref |
| **Moderate CVH** | 0.622(0.536,0.720) | <0.0001 | 0.732(0.622,0.860) | <0.001 | 0.835(0.702,0.994) | 0.043 |
| **High CVH** | 0.517(0.327,0.819) | 0.005 | 0.773(0.504,1.185) | 0.238 | 0.932(0.599,1.449) | 0.753 |
| **P for trend** |  | <0.0001 |  | <0.001 |  | 0.068 |

The crude model did not adjust for any covariates; model 1 adjusted for age, sex, race/ethnicity; and model 2 additionally adjusted for PIR, education level, marital status, alcohol consumption, history of CVD, CKD, and depression from model 1.
