# Supplementary material for: Adherence to higher Life’s Essential 8 scores is linearly associated with reduced all-cause and cardiovascular mortality among US adults with metabolic syndrome: Results from NHANES 2005–2018
Source: PLoS One. 2024 Nov 22;19(11):e0314152. doi: 10.1371/journal.pone.0314152 (PMC11584117; doi:10.1371/journal.pone.0314152)
Supplement: S4 Table — The crude model did not adjust for any covariates; model 1 adjusted for age, sex, race/ethnicity; and model 2 additionally adjusted for PIR, education level, marital status, alcohol consumption, history of CVD, CKD, and depression from model 1. (DOCX) [file pone.0314152.s004.docx]

**S4 Table. Association of LE8 with CVD mortality in the IDF-MetS population.**

|  | **Crude Model**  **HR (95%CI)** | **P-value** | **Model 1**  **HR (95%CI)** | **P-value** | **Model 2**  **HR (95%CI)** | **P-value** |
| --- | --- | --- | --- | --- | --- | --- |
| **LE8** | 0.967(0.957,0.977) | <0.0001 | 0.959(0.947,0.972) | <0.0001 | 0.969(0.957,0.982) | <0.0001 |
| **LE8** | | | | | | |
| **Low CVH** | ref | ref | ref | ref | ref | ref |
| **Moderate CVH** | 0.445(0.330,0.600) | <0.0001 | 0.409(0.297,0.563) | <0.0001 | 0.520(0.376,0.719) | <0.0001 |
| **High CVH** | 0.306(0.133,0.701) | 0.005 | 0.246(0.112,0.540) | <0.001 | 0.383(0.165,0.888) | 0.025 |
| **P for trend** |  | <0.0001 |  | <0.0001 |  | <0.0001 |
| **health behaviors** | 0.987(0.981,0.994) | <0.001 | 0.978(0.971,0.985) | <0.0001 | 0.984(0.976,0.991) | <0.0001 |
| **health behaviors** | | | | | | |
| **Low CVH** | ref | ref | ref | ref | ref | ref |
| **Moderate CVH** | 0.731(0.556,0.960) | 0.024 | 0.558(0.429,0.727) | <0.0001 | 0.690(0.508,0.936) | 0.017 |
| **High CVH** | 0.472(0.308,0.722) | <0.001 | 0.331(0.216,0.506) | <0.0001 | 0.446(0.287,0.693) | <0.001 |
| **P for trend** |  | <0.001 |  | <0.0001 |  | <0.001 |
| **health factors** | 0.974(0.965,0.983) | <0.0001 | 0.978(0.968,0.989) | <0.0001 | 0.985(0.974,0.995) | 0.004 |
| **health factors** | | | | | | |
| **Low CVH** | ref | ref | ref | ref | ref | ref |
| **Moderate CVH** | 0.501(0.385,0.652) | <0.0001 | 0.589(0.442,0.785) | <0.001 | 0.668(0.496,0.899) | 0.008 |
| **High CVH** | 0.441(0.213,0.913) | 0.028 | 0.657(0.336,1.286) | 0.22 | 0.807(0.406,1.606) | 0.541 |
| **P for trend** |  | <0.0001 |  | <0.001 |  | 0.017 |

The crude model did not adjust for any covariates; model 1 adjusted for age, sex, race/ethnicity; and model 2 additionally adjusted for PIR, education level, marital status, alcohol consumption, history of CVD, CKD, and depression from model 1.
