# Supplementary material for: Adherence to higher Life’s Essential 8 scores is linearly associated with reduced all-cause and cardiovascular mortality among US adults with metabolic syndrome: Results from NHANES 2005–2018
Source: PLoS One. 2024 Nov 22;19(11):e0314152. doi: 10.1371/journal.pone.0314152 (PMC11584117; doi:10.1371/journal.pone.0314152)
Supplement: S5 Table — The crude model did not adjust for any covariates; model 1 adjusted for age, sex, race/ethnicity; and model 2 additionally adjusted for PIR, education level, marital status, alcohol consumption, history of CVD, CKD, and depression from model 1. (DOCX) [file pone.0314152.s005.docx]

**S5 Table. Association of LE8 with cancer mortality in the IDF-MetS population.**

|  | **Crude Model**  **HR (95%CI)** | **P-value** | **Model 1**  **HR (95%CI)** | **P-value** | **Model 2**  **HR (95%CI)** | **P-value** |
| --- | --- | --- | --- | --- | --- | --- |
| **LE8** | 0.990(0.978,1.002) | 0.114 | 0.987(0.973,1.000) | 0.056 | 0.995(0.981,1.009) | 0.464 |
| **LE8** | | | | | | |
| **Low CVH** | ref | ref | ref | ref | ref | ref |
| **Moderate CVH** | 0.917(0.617,1.363) | 0.668 | 0.867(0.576,1.304) | 0.492 | 1.027(0.683,1.545) | 0.896 |
| **High CVH** | 0.470(0.112,1.969) | 0.301 | 0.408(0.097,1.719) | 0.222 | 0.562(0.126,2.518) | 0.452 |
| **P for trend** |  | 0.326 |  | 0.187 |  | 0.727 |
| **health behaviors** | 0.995(0.986,1.003) | 0.237 | 0.988(0.979,0.997) | 0.012 | 0.993(0.984,1.002) | 0.138 |
| **health behaviors** | | | | | | |
| **Low CVH** | ref | ref | ref | ref | ref | ref |
| **Moderate CVH** | 1.152(0.816,1.625) | 0.421 | 0.938(0.658,1.336) | 0.721 | 1.131(0.792,1.616) | 0.499 |
| **High CVH** | 0.843(0.475,1.496) | 0.56 | 0.630(0.360,1.103) | 0.106 | 0.827(0.473,1.447) | 0.506 |
| **P for trend** |  | 0.563 |  | 0.084 |  | 0.489 |
| **health factors** | 0.995(0.983,1.006) | 0.369 | 1.001(0.989,1.014) | 0.846 | 1.004(0.992,1.017) | 0.517 |
| **health factors** | | | | | | |
| **Low CVH** | ref | ref | ref | ref | ref | ref |
| **Moderate CVH** | 0.884(0.621,1.259) | 0.495 | 1.021(0.714,1.459) | 0.91 | 1.105(0.763,1.599) | 0.598 |
| **High CVH** | 0.860(0.355,2.082) | 0.738 | 1.271(0.518,3.118) | 0.6 | 1.404(0.565,3.490) | 0.465 |
| **P for trend** |  | 0.485 |  | 0.703 |  | 0.425 |

The crude model did not adjust for any covariates; model 1 adjusted for age, sex, race/ethnicity; and model 2 additionally adjusted for PIR, education level, marital status, alcohol consumption, history of CVD, CKD, and depression from model 1.
