# Supplementary material for: Adherence to higher Life’s Essential 8 scores is linearly associated with reduced all-cause and cardiovascular mortality among US adults with metabolic syndrome: Results from NHANES 2005–2018
Source: PLoS One. 2024 Nov 22;19(11):e0314152. doi: 10.1371/journal.pone.0314152 (PMC11584117; doi:10.1371/journal.pone.0314152)
Supplement: S6 Table — (DOCX) [file pone.0314152.s006.docx]

**S6 Table. Association of LE8, health behaviors, and health factors with all-cause mortality in the MetS population after excluding participants with chronic kidney disease, cardiovascular disease, cancer, or depression at baseline.**

| **ALL** | **Crude Model**  **HR (95%CI)** | **P-value** | **Model 1**  **HR (95%CI)** | **P-value** | **Model 2**  **HR (95%CI)** | **P-value** |
| --- | --- | --- | --- | --- | --- | --- |
| **LE8** | 0.977(0.964,0.991) | <0.001 | 0.977(0.963,0.990) | <0.001 | 0.983(0.969,0.997) | 0.015 |
| **LE8** | | | | | | |
| <50 | ref | ref | ref | ref | ref | ref |
| 50-80 | 0.544(0.369,0.802) | 0.002 | 0.537(0.362,0.797) | 0.002 | 0.621(0.411,0.938) | 0.024 |
| >80 | 0.246(0.084,0.725) | 0.011 | 0.228(0.082,0.638) | 0.005 | 0.287(0.099,0.836) | 0.022 |
| P for trend |  | <0.001 |  | <0.001 |  | 0.004 |
| **health behaviors** | 0.992(0.983,1.001) | 0.068 | 0.985(0.976,0.993) | <0.001 | 0.989(0.980,0.998) | 0.013 |
| **health behaviors** | | | | | | |
| <50 | ref | ref | ref | ref | ref | ref |
| 50-80 | 0.776(0.528,1.140) | 0.197 | 0.601(0.425,0.849) | 0.004 | 0.662(0.459,0.955) | 0.027 |
| >80 | 0.728(0.439,1.210) | 0.221 | 0.502(0.313,0.805) | 0.004 | 0.615(0.376,0.997) | 0.043 |
| P for trend |  | 0.236 |  | 0.008 |  | 0.002 |
| **health factors** | 0.981(0.970,0.992) | <0.001 | 0.992(0.979,1.004) | 0.178 | 0.994(0.982,1.006) | 0.318 |
| **health factors** | | | | | | |
| <50 | ref | ref | ref | ref | ref | ref |
| 50-80 | 0.579(0.427,0.785) | <0.001 | 0.738(0.543,1.004) | 0.053 | 0.782(0.575,1.063) | 0.116 |
| >80 | 0.521(0.225,1.209) | 0.129 | 0.957(0.407,2.249) | 0.919 | 1.006(0.414,2.445) | 0.99 |
| P for trend |  | 0.002 |  | 0.167 |  | 0.296 |
