# Supplementary material for: Adherence to higher Life’s Essential 8 scores is linearly associated with reduced all-cause and cardiovascular mortality among US adults with metabolic syndrome: Results from NHANES 2005–2018
Source: PLoS One. 2024 Nov 22;19(11):e0314152. doi: 10.1371/journal.pone.0314152 (PMC11584117; doi:10.1371/journal.pone.0314152)
Supplement: S7 Table — (DOCX) [file pone.0314152.s007.docx]

**S7 Table. Association of LE8, health behaviors, and health factors with CVD mortality in the MetS population after excluding participants with chronic kidney disease, cardiovascular disease, cancer, or depression at baseline.**

| **CVD** | **Crude Model**  **HR (95%CI)** | **P-value** | **Model 1**  **HR (95%CI)** | **P-value** | **Model 2**  **HR (95%CI)** | **P-value** |
| --- | --- | --- | --- | --- | --- | --- |
| **LE8** | 0.966(0.956,0.977) | <0.0001 | 0.962(0.950,0.974) | <0.0001 | 0.967(0.954,0.980) | <0.0001 |
| **LE8** | | | | | | |
| <50 | ref | ref | ref | ref | ref | ref |
| 50-80 | 0.449(0.334,0.604) | <0.0001 | 0.443(0.327,0.601) | <0.0001 | 0.488(0.356,0.670) | <0.0001 |
| >80 | 0.273(0.117,0.635) | 0.003 | 0.242(0.109,0.537) | <0.001 | 0.311(0.137,0.709) | 0.005 |
| P for trend |  | <0.0001 |  | <0.0001 |  | <0.0001 |
| **health behaviors** | 0.987(0.980,0.994) | <0.001 | 0.981(0.973,0.989) | <0.0001 | 0.984(0.976,0.993) | <0.001 |
| **health behaviors** | | | | | | |
| <50 | ref | ref | ref | ref | ref | ref |
| 50-80 | 0.729(0.547,0.971) | 0.031 | 0.601(0.455,0.795) | <0.001 | 0.657(0.489,0.882) | 0.005 |
| >80 | 0.530(0.339,0.827) | 0.005 | 0.413(0.261,0.654) | <0.001 | 0.482(0.303,0.768) | 0.002 |
| P for trend |  | 0.003 |  | <0.0001 |  | 0.002 |
| **health factors** | 0.973(0.964,0.982) | <0.0001 | 0.978(0.968,0.988) | <0.0001 | 0.980(0.969,0.990) | <0.001 |
| **health factors** | | | | | | |
| <50 | ref | ref | ref | ref | ref | ref |
| 50-80 | 0.510(0.390,0.667) | <0.0001 | 0.602(0.457,0.793) | <0.001 | 0.628(0.474,0.832) | 0.001 |
| >80 | 0.412(0.190,0.892) | 0.025 | 0.600(0.296,1.218) | 0.157 | 0.668(0.324,1.380) | 0.276 |
| P for trend |  | <0.0001 |  | <0.001 |  | 0.003 |
