# Supplementary material for: Adherence to higher Life’s Essential 8 scores is linearly associated with reduced all-cause and cardiovascular mortality among US adults with metabolic syndrome: Results from NHANES 2005–2018
Source: PLoS One. 2024 Nov 22;19(11):e0314152. doi: 10.1371/journal.pone.0314152 (PMC11584117; doi:10.1371/journal.pone.0314152)
Supplement: S8 Table — Model 1: Age, Sex, Race. Model 2: Age, Sex, Race, PIR, marital status, education, drinking. (DOCX) [file pone.0314152.s008.docx]

**S8 Table. Association of LE8, health behaviors, and health factors with cancer mortality in the MetS population after excluding participants with chronic kidney disease, cardiovascular disease, cancer, or depression at baseline.**

| **Cancer** | **Crude Model**  **HR (95%CI)** | **P-value** | **Model 1**  **HR (95%CI)** | **P-value** | **Model 2**  **HR (95%CI)** | **P-value** |
| --- | --- | --- | --- | --- | --- | --- |
| **LE8** | 0.985(0.972,0.999) | 0.032 | 0.984(0.969,0.998) | 0.031 | 0.984(0.970,1.001) | 0.051 |
| **LE8** | | | | | | |
| <50 | ref | ref | ref | ref | ref | ref |
| 50-80 | 0.789(0.522,1.193) | 0.261 | 0.779(0.509,1.190) | 0.247 | 0.785(0.512,1.204) | 0.268 |
| >80 | 0.388(0.102,1.481) | 0.166 | 0.358(0.094,1.366) | 0.133 | 0.374(0.097,1.445) | 0.154 |
| P for trend |  | 0.103 |  | 0.081 |  | 0.101 |
| **health behaviors** | 0.990(0.981,0.999) | 0.022 | 0.985(0.976,0.994) | <0.001 | 0.985(0.976,0.994) | 0.002 |
| **health behaviors** | | | | | | |
| <50 | ref | ref | ref | ref | ref | ref |
| 50-80 | 0.974(0.659,1.439) | 0.894 | 0.829(0.558,1.232) | 0.354 | 0.837(0.561,1.249) | 0.383 |
| >80 | 0.675(0.394,1.157) | 0.153 | 0.540(0.322,0.904) | 0.019 | 0.549(0.326,0.924) | 0.024 |
| P for trend |  | 0.138 |  | 0.015 |  | 0.019 |
| **health factors** | 0.996(0.985,1.007) | 0.509 | 1.003(0.991,1.015) | 0.64 | 1.003(0.991,1.015) | 0.611 |
| **health factors** | | | | | | |
| <50 | ref | ref | ref | ref | ref | ref |
| 50-80 | 0.978(0.699,1.369) | 0.896 | 1.132(0.805,1.592) | 0.476 | 1.136(0.806,1.602) | 0.465 |
| >80 | 0.970(0.428,2.194) | 0.941 | 1.400(0.614,3.194) | 0.424 | 1.431(0.626,3.269) | 0.396 |
| P for trend |  | 0.889 |  | 0.327 |  | 0.31 |

Model 1: Age, Sex, Race

Model 2: Age, Sex, Race, PIR, marital status, education, drinking
