# Supplementary material for: Adherence to higher Life’s Essential 8 scores is linearly associated with reduced all-cause and cardiovascular mortality among US adults with metabolic syndrome: Results from NHANES 2005–2018
Source: PLoS One. 2024 Nov 22;19(11):e0314152. doi: 10.1371/journal.pone.0314152 (PMC11584117; doi:10.1371/journal.pone.0314152)

**S1 Fig. Stratified analysis of the association between LE8 and cancer mortality in the MetS population.**


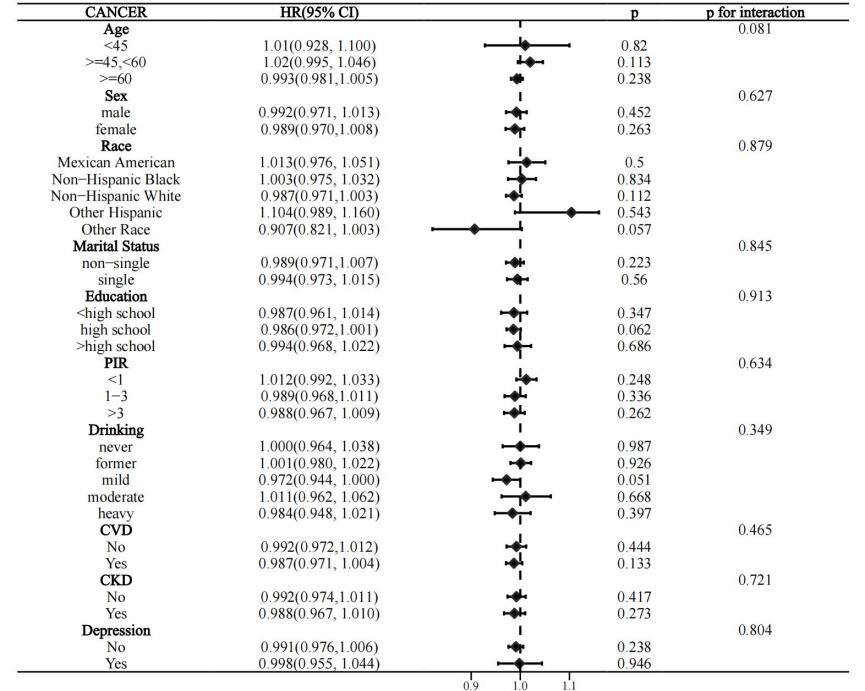

Supplement: S1 Fig — (DOCX) [file pone.0314152.s009.docx]
